# Imagining tomorrow's university in an era of open science

**DOI:** 10.12688/f1000research.11232.2

**Published:** 2017-05-19

**Authors:** Adina Howe, Michael Howe, Amy L. Kaleita, D. Raj Raman

**Affiliations:** 1 Department of Agricultural and Biosystems Engineering, Iowa State University, Ames, IA, 50011, USA; 2 Department of Management, Iowa State University, Ames, IA, 50011, USA

**Keywords:** open science, university, education, training, data, impact

## Abstract

As part of a recent workshop entitled "Imagining Tomorrow's University”, we were asked to visualize the future of universities as research becomes increasingly data- and computation-driven, and identify a set of principles characterizing pertinent opportunities and obstacles presented by this shift. In order to establish a holistic view, we take a multilevel approach and examine the impact of open science on individual scholars and how this impacts as well as on the university as a whole. At the university level, open science presents a double-edged sword: when well executed, open science can accelerate the rate of scientific inquiry across the institution and beyond; however, haphazard or half-hearted efforts are likely to squander valuable resources, diminish university productivity and prestige, and potentially do more harm than good. We present our perspective on the role of open science at the university.

## Introduction

The mission of universities, specifically land-grant institutions originating from the Morrill Act of 1862, is to provide accessible education and scholarship to *all* people. In a similar vein, open science has emerged as an approach to minimize the barriers associated with traditional ways of sharing the outcomes of scholarship. As defined by the Open Definition ( https://okfn.org/), open science embodies the notion that information is available for anyone to “freely access, use, modify, and share for any purpose”, regardless of personal or institutional resources. Fostered by increasingly data- and computation-driven research, universities are uniquely positioned to reimagine their role in knowledge dissemination vis-à-vis the principles of open science. As part of a recent workshop entitled “ Imagining Tomorrow's University”, we were asked to visualize the future of universities in an open, networked era and to identify a set of principles characterizing pertinent opportunities and obstacles presented by this shift. In order to establish a holistic view, we take a multilevel approach and examine the impact of open science on individual scholars as well as on the university as a whole. Generally, we agree that increased transparency in the scientific process can broaden and deepen scientific inquiry, understanding, and impact. However, the realization of these outcomes will require significant time, effort, and aptitude to successfully convey the means by which data are transformed into knowledge. We propose that open science can most effectively enable this evolution when it is conceptualized as a multifaceted pathway that includes:

- The provision of accessible and well-described data, along with information about its context ^1^;
- The methodology and mechanisms necessary to reproduce data analyses;
- Training products that provide transparent understanding of how the data can be applied to answer questions.

Thus, impactful open science requires investments from individual researchers that are often greater than those that might be needed for “non-open” science. At the university level, open science represents a double-edged sword: when well executed, it can accelerate the rate of scientific inquiry across the institution and beyond; however, haphazard or half-hearted efforts are likely to squander valuable resources and diminish university productivity and prestige, potentially doing more harm than good. Here, we present our perspective on the varying roles of open science.

## Open science enables low-barrier collaborations

For some university researchers, open science can be both powerful and transformative ^2^. Imagine a research program that generates not only publications but also develops code that can quickly reproduce each analysis and publishable figure with a minimal amount of manual intervention. This structure can provide continuity in a project and accelerate the research enterprise by allowing researchers to rapidly repeat the same analysis on new datasets, all while lowering training and other human capital investments. Included in a publication, this “research notebook” and accompanying datasets (e.g., 3), could be compiled into a tutorial for others in the field who could then repeat this work with their own data – all without the need for formal collaborations. Such approaches can benefit not only the initiating research group but also an entire scientific discipline. **

## Open science imposes significant costs

While the opportunities of open science practices hold promise, several costs and obstacles may prevent its realization and impact. A key cost of open science is time – time to format, annotate and publish data and associated metadata; time to learn new tools that allow for automated analysis and reproduction; and time to produce scripts with a sufficient level of robustness and documentation to be broadly useful to others ^4^. Of these, arguably, the least time-consuming step is simply providing access to data. While open data is an important component of open science, it requires significant investment and does not provide the broad benefits of open science writ large. These investments include personnel, labor, computational infrastructure to store data, and publication costs to communicate findings in an open way (up to $3900, 5). Consequently, a challenge for university engagement with open science is determining who should bear the financial burden of these costs. For example, a grant proposal that requires $10,000 additional for open access publishing fees may not be viewed as competitively as one with these dollars allocated for direct research costs. Similarly, while universities can directly promote open science by subsidizing open access publication fees or providing cost-sharing opportunities, they too must decide where best to invest their limited resources.

Further, it would be irresponsible to discuss open data and open science without acknowledging the risk posed to the anonymity that is so central to many human research studies. For example, to promote participant anonymity, data resulting from research currently conducted under the auspices of an IRB may be ineligible for distribution outside of the immediate research team. As multiple sources of open data become increasingly available, privacy concerns of this nature are likely to increase along with the prevalence of unintended participant identification ^6, 7^. In these cases, the benefits of open science may not stem from sharing data but rather reproducible analyses that may be more broadly useful, and the provision of open data does not in itself translate into our vision of open science. At the university level, the incentives to facilitate and expand open science at the university should not be monolithic (e.g., data-centric), but rather be selectively created and applied to maximize success and minimize unintended harm. Open science also presents unique challenges as universities and other research institutions turn increasingly to private sector funding, which comes with proprietary limitations on the dissemination of results.

## The potential for broader impacts with open science

It is possible that the increasing availability and transparency of scientific inquiry could ignite broader interest in research. The current publishing paradigm of most fields limits research availability to a relatively narrow audience, with paid access to scientific journals. Meanwhile, polling data from Gallup indicates a slow but relatively steady decline in Americans’ trust of institutions in general since 2000 ^8^, although Gallup does not include “universities” specifically in the poll. In one study that compared follow-on inventions from discoveries that were made simultaneously but separately at a university and at a corporate firm, the same discovery at a university was 20–30% less likely to be used in follow-up innovations ^9^. This study also included open-ended interviews to shed light on this “Ivory Tower effect”, and a driver appeared to be “considerable skepticism toward academic science.” More openness in university science research may help to address this apparent skepticism.

Even though there are concerns associated with society’s growing disconnect with the scientific enterprise and the accompanying devaluation of research, it should be noted that in general academics are still held in high regard and seen as reliable sources of information for a wide range of issues ^10, 11^. To maintain this esteem, it is important to realize that data without an understanding of what it entails or the questions it can answer can be considered useless and even dangerous when used improperly to influence decision-making and policy ^12^. Thus, providing useful open data requires more thought on how this data can be translated into useful information. Mechanisms to reproduce analyses and communications that explain the complexities and intricacies of these tasks could be an important first step. While the peer-reviewed-publication paradigm currently provides an established, if not optimal, communication mechanism for conveying the results of scientific activities to our peers, no such standard currently exists to govern the creation and exchange of open science to our peers and beyond. Efforts at the university level that encourage the rigorous construction of appropriate dissemination systems are laying the foundation for success in this endeavor.

## A path forward: recognition, training and infrastructure

*Recognize open science impacts.* Universities have a moral responsibility to educate, and there are significant opportunities in the open science model to broaden the output of research with an eye towards education. Nevertheless, the current university promotion and tenure system is optimized for evaluating the traditional format of peer-reviewed journals as the only necessary and sufficient product of a research project. Given the “publish or perish” paradigm that currently pervades the academy, an accompanying lack of recognition for the time and effort put into facilitating open science is apt to dampen participation ^12^. For example, utilizing openly available code for an analysis in a subsequent publication does not require a citation, and even if the code were to be highly cited, it does not carry the same weight as a peer-reviewed publication. Thus, universities have an opportunity to re-imagine what it means to contribute to research, specifically extending the definition to include more than a tally of peer reviewed publications. The development of robust, reliable, and transparent tools to track utilization of open science products may be one path forward to quantitatively measure the impact of faculty generated research outputs not currently tracked or rewarded, and both incentivize and acknowledge the resources required to effectively engage in open science.

*Train best practices and provide infrastructure to broaden participation.* A notable effort to define the characteristics of open science products are the FAIR Data Principles ^13^, which emphasize that scholarly products should be findable, accessible, interoperable, and reusable and that good data management is not a goal in itself but can catalyze knowledge discovery and innovation. At the university, training for sustainable data management best practices would deepen the overall understanding of the opportunities inherent in open science. In many respects, the products of open science are available to benefit by all that require support infrastructure to share data, tools, and training to broaden participation and limit exploitation. This infrastructure could also be re-imagined to include metrics to quantify impact, supporting the need to acknowledge contributions.

In conclusion, open science is a significant opportunity for universities, but a one-size-fits-all approach is sub-optimal. Executing open science in a way that facilitates meaningful advances requires a personal investment of time, both upfront to develop relevant capabilities, and ongoing for execution expenses. As such, it is important that universities develop infrastructure and training to support, measure, and reward efforts that deliver on the promise of open science, focusing on domains best positioned to further scientific understanding.

A preprint of this article can be found on PeerJ ( https://doi.org/10.7287/peerj.preprints.2781v1).
