# Supplementary material for: Imagining tomorrow's university in an era of open science
Source: F1000Res. 2017 May 19;6:405. Originally published 2017 Mar 31. [Version 2] doi: 10.12688/f1000research.11232.2 (PMC5437947; doi:10.12688/f1000research.11232.2)
Supplement: Peer review history

# Peer review: Referee response for version 2

**DOI:** 10.5256/f1000research.12634.r22930

**Published:** 2017-05-22

**Authors:** Nathan L. Vanderford

**Affiliations:** 1 Markey Cancer Center, Department of Toxicology and Cancer Biology, College of Medicine, University of Kentucky, Lexington, KY, USA

Howe et al. have been responsive to my previous review as well as to the review of the other two reviewers. The article is improved and I find it to be acceptable at this stage.

I have read this submission. I believe that I have an appropriate level of expertise to confirm that it is of an acceptable scientific standard.

# Peer review: Referee response for version 1

**DOI:** 10.5256/f1000research.12118.r22286

**Published:** 2017-05-15

**Authors:** Marie-Claire Shanahan

**Affiliations:** 1 University of Calgary, Calgary, AB, Canada

This is a timely and important topic, and it is clear that the authors have first-hand experience with the area of open science related to creating open and shared analysis code (e.g., Citation #2). They have a very valuable perspective to add to the scientific community's conversations about open science. And while I definitely support the eventual indexing of this article, I feel that there are some areas in which the argument should be strengthened and clarified first.

 As reviewer Vanderford notes, a clear definition of open science is needed early on. This would also be very helpful for strengthening the arguments that develop in the middle and final sections that open databases are not sufficient for open science and that universities need better infrastructure for recognizing shared code as an academic contribution. A clear definition would also help place the open analysis code argument more clearly. Is sharing analysis programs and code sufficient for open science (i.e., is it synonymous with open science) or is it instead an under-recognized but important element or type of open science that the authors wish to highlight?

 The paper seems to settle in on open analysis code as the central argument later on, but in the opening sections the argument seems overly broad for the examples and support that are given. The only example of benefits that is given is of the "open notebook" (a good and valuable example) but it is not sufficient to support broad claims about open science as a double-edged sword (where neither the broads benefits or potential downfalls are explained in detail or supported with evidence from the literature). I think focusing the argument and placing their perspective more clearly within the broader field of open science early on would create a more cohesive argument and one that can be better supported with the experiences and examples the authors provide.

*Is the topic of the opinion article discussed accurately in the context of the current literature?*

*Are all factual statements correct and adequately supported by citations?*

*Are arguments sufficiently supported by evidence from the published literature?*

 In addition to the example above there are a few places where support for statements and accuracy with relation to the literature could be improved. Defining open science clearly and placing the authors' perspectives related to open analysis code within that larger definition would help improve connections to the literature. A few examples are below that might be helpful.

 Later on, citations 7 and 8 are used to support discussion of take up of academic discoveries. These are both citations of the same study though, with 7 being the study itself and 8 being a popular media report on the study. Using both seems to suggest that there are two independent sources to support this claim. There is also a published version of the study that might be a preferred citation ^1^. And I think it might be helpful to note that the study does not find mistrust to be the main reason for lack of uptake but says it is secondary to the natural competitiveness of industrial science, where they are constantly monitoring competitors and therefore likely to notice discoveries that competitors make.

 "To maintain this esteem, it is important to realize that data without an understanding of what it entails or the questions it can answer can be considered useless and even dangerous when used improperly to influence decision making and policy" [11]. This is a strong claim, and it could represent important reservations about open science, but no support is provided. The citation does not seem to be related at all (Title: "Electrically conductive bacterial nanowires produced by Shewanella oneidensis strain MR-1 and other microorganisms.") Are the authors referring to some controversy surrounding the inappropriate use of that data for decision making and policy? If so, this needs to be explained and supported explicitly. Otherwise, other citations should be found to support this claim.

 Referring to Hardin's "Tragedy of the commons" [Citation 14] also does not seem like a closely related source for the use of the term "common good" as the authors have used it. Some clarification would be helpful there as well. Neilsen's book ^2^, similarly talks about "knowledge commons" specifically in relation to open science in a way that might be more relevant to the arguments made here.

*Are the conclusions drawn balanced and justified on the basis of the presented arguments?*

 In the end, the authors make an important and valid argument about university supports and infrastructure, but the points leading up to that conclusion could be more clearly explained and better supported and connected. From the section "A path forward" and onward, the examples lead nicely towards the conclusion. Given the authors expertise I feel that the could be expanded a bit and to explore and support the conclusion, and the earlier paragraphs could focus more specifically on the issues of open analysis code to build towards that conclusion. For example, in discussing the Ivory Tower effect earlier on, the original study that is cited explores the publish or perish system as one of the reasons for distrust. The argument made about publish or perish later on could be more meaningful if that connection had been made in the previous section.

 Overall, the authors have an important contribution to make to discussions of open science and important expertise in the practice of open science. With some clarification to the supports and the argument, this paper will be a valuable and interesting piece of that conversation.

I have read this submission. I believe that I have an appropriate level of expertise to confirm that it is of an acceptable scientific standard, however I have significant reservations, as outlined above.

## References

[1] BikardM: Is Academic Science Trapped Inside the Ivory Tower? Universities and the Diffusion of New Knowledge. *Academy of Management Proceedings*.2012;2012(1) : 10.5465/AMBPP.2012.189 10.5465/AMBPP.2012.189

[2] NielsenM: Reinventing discovery: the new era of networked science. *Princeton University Press*.2012;

[3] NosekBAAlterGBanksGCBorsboomDBowmanSDBrecklerSJBuckSChambersCDChinGChristensenGContestabileMDafoeAEichEFreeseJGlennersterRGoroffDGreenDPHesseBHumphreysMIshiyamaJKarlanDKrautALupiaAMabryPMadonTAMalhotraNMayo-WilsonEMcNuttMMiguelEPaluckELSimonsohnUSoderbergCSpellmanBATurittoJVandenBosGVazireSWagenmakersEJWilsonRYarkoniT: SCIENTIFIC STANDARDS. Promoting an open research culture. *Science*.2015;348(6242) : 10.1126/science.aab2374 1422-5 10.1126/science.aab2374 26113702PMC4550299

[4] EasterbrookS: Open code for open science?. *Nature Geoscience*.2014;7(11) : 10.1038/ngeo2283 779-781 10.1038/ngeo2283

# Response

**Published:** 2017-05-17

**Authors:** Adina Howe

**Affiliations:** Iowa State University, USA

1.  As reviewer Vanderford notes, a clear definition of open science is needed early on. This would also be very helpful for strengthening the arguments that develop in the middle and final sections that open databases are not sufficient for open science and that universities need better infrastructure for recognizing shared code as an academic contribution. A clear definition would also help place the open analysis code argument more clearly. Is sharing analysis programs and code sufficient for open science (i.e., is it synonymous with open science) or is it instead an under-recognized but important element or type of open science that the authors wish to highlight?

 Thank you for this suggestion.  We have now included a clearer open science definition within the introduciton to help clarify our perspective and define “sharing” data as an important element within open access themes of  “access, use, modify, and sharing”.

 2.  The paper seems to settle in on open analysis code as the central argument later on, but in the opening sections the argument seems overly broad for the examples and support that are given. The only example of benefits that is given is of the "open notebook" (a good and valuable example) but it is not sufficient to support broad claims about open science as a double-edged sword (where neither the broads benefits or potential downfalls are explained in detail or supported with evidence from the literature). I think focusing the argument and placing their perspective more clearly within the broader field of open science early on would create a more cohesive argument and one that can be better supported with the experiences and examples the authors provide.

 In response to reviewer #2, more specific examples, of the benefits and barriers to open science have been included to represent the broader field of open science (e.g. publishing cost).  Further, we believe that the modification of the title and introduction revisions help to focus the central topic of this effort on the impacts of a university in an open era.  Finally, for more specific examples, we have also cited a McKiernan et al. 2016 which represents a more data-centric approach of the benefits of open science.

 3.  In addition to the example above there are a few places where support for statements and accuracy with relation to the literature could be improved. Defining open science clearly and placing the authors' perspectives related to open analysis code within that larger definition would help improve connections to the literature. A few examples are below that might be helpful.

 Later on, citations 7 and 8 are used to support discussion of take up of academic discoveries. These are both citations of the same study though, with 7 being the study itself and 8 being a popular media report on the study. Using both seems to suggest that there are two independent sources to support this claim. There is also a published version of the study that might be a preferred citation ^1^. And I think it might be helpful to note that the study does not find mistrust to be the main reason for lack of uptake but says it is secondary to the natural competitiveness of industrial science, where they are constantly monitoring competitors and therefore likely to notice discoveries that competitors make.

 Thank you for these comments – we agree that two citations here is misleading and have removed the citation #8 and replaced the original preprint with the suggested citation provided more recent published study (https://papers.ssrn.com/sol3/papers.cfm?abstract_id=2333413).  The general results of the cited study indicate that “the results suggest that the peer-based knowledge validation process in academia creates uncertainty about the reliability and relevance of academic science as a map for technology development.” [Bikard, 2015].  We also modified the reason inventors draw on knowledge from firms rather than academics to be associated (e.g., a driver vs a key driver) with skepticism toward academic science.

 4.  "To maintain this esteem, it is important to realize that data without an understanding of what it entails or the questions it can answer can be considered useless and even dangerous when used improperly to influence decision making and policy" [11]. This is a strong claim, and it could represent important reservations about open science, but no support is provided. The citation does not seem to be related at all (Title: "Electrically conductive bacterial nanowires produced by Shewanella oneidensis strain MR-1 and other microorganisms.") Are the authors referring to some controversy surrounding the inappropriate use of that data for decision making and policy? If so, this needs to be explained and supported explicitly. Otherwise, other citations should be found to support this claim.

 Thank you for this observation – we’ve corrected the citation.

 5.  Referring to Hardin's "Tragedy of the commons" [Citation 14] also does not seem like a closely related source for the use of the term "common good" as the authors have used it. Some clarification would be helpful there as well. Neilsen's book ^2^, similarly talks about "knowledge commons" specifically in relation to open science in a way that might be more relevant to the arguments made here.

 To clarify this phrase, we modified “common good” to explicitly state “available to benefit by all”.

**

 6.  In the end, the authors make an important and valid argument about university supports and infrastructure, but the points leading up to that conclusion could be more clearly explained and better supported and connected. From the section "A path forward" and onward, the examples lead nicely towards the conclusion. Given the authors expertise I feel that the could be expanded a bit and to explore and support the conclusion, and the earlier paragraphs could focus more specifically on the issues of open analysis code to build towards that conclusion. For example, in discussing the Ivory Tower effect earlier on, the original study that is cited explores the publish or perish system as one of the reasons for distrust. The argument made about publish or perish later on could be more meaningful if that connection had been made in the previous section.

 Thank you for your suggestions.  With these suggestions in mind, we have edited the text for a more natural flow, using both additional examples and citations and specific headers.

# Peer review: Referee response for version 1

**DOI:** 10.5256/f1000research.12118.r22285

**Published:** 2017-04-27

**Authors:** Nathan L. Vanderford

**Affiliations:** 1 Markey Cancer Center, Department of Toxicology and Cancer Biology, College of Medicine, University of Kentucky, Lexington, KY, USA

This article by Howe et al. is a generally well-written commentary on the importance of open science as well as some of the current barriers to its most effective implementation and use. This is a very timely topic that certainly deserves discussion of its value to the scientific community and of how it can be improved.

 I believe there are several noteworthy areas in which the article could be improved prior to it being approved for indexing.

1. The authors should step back at the very onset of the article and define open science. It would also be helpful to provide more context on the history of open science and why/ how that history is important to the current article.
2. While this is a well-written article in general, there are a few phrases used that should be reconsidered. For example, the use of the phrase “the whole enchilada” should be re-written in a more professional phrasing.
3. I would argue that the most significant “costs” or “barriers” to open science are the financial costs (of which personnel labor/ time and electronic/ computer storage space issues are perhaps the biggest components). It is a missed opportunity to not mention the financial costs of open science. As part of that discussion, it would be interesting for the authors to discuss who bears the financial costs and whether there is room for improvement. For example, could funders/sponsors do more to support the costs of open science? And, are there opportunities for new policies (at the level of funders/sponsors) to be developed that could further support the wider implementation of open science?
4. The title of the article refers to the “impact” of open science but the current content of the article falls short on convincing the reader of the current and potential impact of open science. It may be worth providing some specific examples of how open science has been used to make impactful discoveries, etc. Providing such examples could drive home the point of why it is so important to support and improve open science. 

In summary, this is a timely article discussing a very important topic. There are limitations of the current version of the article that dampen this reviewer’s enthusiasm at this time regarding giving a full approval. As such, I look forward to reviewing a revised version of the article.

I have read this submission. I believe that I have an appropriate level of expertise to confirm that it is of an acceptable scientific standard, however I have significant reservations, as outlined above.

# Response

**Published:** 2017-05-17

**Authors:** Adina Howe

**Affiliations:** Iowa State University, USA

1. The authors should step back at the very onset of the article and define open science. It would also be helpful to provide more context on the history of open science and why/ how that history is important to the current article.

Great suggestion.  We agree that this was missing and we now provide a context for universities and their role in open science as an introduction.

2. While this is a well-written article in general, there are a few phrases used that should be reconsidered. For example, the use of the phrase “the whole enchilada” should be re-written in a more professional phrasing.

Modified.

3. I would argue that the most significant “costs” or “barriers” to open science are the financial costs (of which personnel labor/ time and electronic/ computer storage space issues are perhaps the biggest components). It is a missed opportunity to not mention the financial costs of open science. As part of that discussion, it would be interesting for the authors to discuss who bears the financial costs and whether there is room for improvement. For example, could funders/sponsors do more to support the costs of open science? And, are there opportunities for new policies (at the level of funders/sponsors) to be developed that could further support the wider implementation of open science?

Thank you for pointing out this opportunity in this piece.  We have added a discussion to bring up the topic of financial costs associated with open science and the complexities of determining incentives at the university level.  While there is opportunity for funders/sponsors to help bear these financial burdens, the scope of this effort is what a university can do, and we have limited our discussion to this topic. 

4. The title of the article refers to the “impact” of open science but the current content of the article falls short on convincing the reader of the current and potential impact of open science. It may be worth providing some specific examples of how open science has been used to make impactful discoveries, etc. Providing such examples could drive home the point of why it is so important to support and improve open science. 

We agree that the title of this article was not a good fit for the content of the piece and have adjusted it accordingly.  We have provided citations to one peer reviewed article that identifies examples and a perspective of how open science have provided impact. A challenge in providing specific examples it that the impact of open science is still debated and difficult to quantify.  Most articles are often personal perspectives of specific authors and not necessarily data-driven approaches to study the positive impacts of open science (which are currently rather limited). Further, there are also examples of negative impacts of open science, http://www.nature.com/news/open-data-contest-unearths-scientific-gems-and-controversy-1.21572?WT.mc_id=TWT_NatureNews.    We hope to present a balanced perspective on the complexities of open science at universities and guide thoughts on the most impactful path forward in this diverse environment.

# Peer review: Referee response for version 1

**DOI:** 10.5256/f1000research.12118.r21433

**Published:** 2017-04-21

**Authors:** May Khanna

**Affiliations:** 1 Department of Pharmacology, College of Medicine, University of Arizona, Tucson, AZ, USA

This was a very well-written manuscript and quite relevant to our current times since there is a definite push for open science in academia. This is becoming increasingly necessary as we continually acquire larger data sets. I have a few comments and suggestions. 

 I would replace the words “the whole enchilada” under the headline “Open science requires significant Investment”. I would also replace “so on” in the same section.

 Instead of the headline “Broader impact of open science is uncertain” may I suggest something along the line of: “Open and broad communication could impact open science”.

 In the section “Open Science requires significant investment”, it is suggested that a key cost of open science is time, which is reasonable. However, one of the points is that “time to produce scripts with a sufficient level of robustness and documentation to be useful to others”; this point is less reasonable. I believe with or without open science, this should an integral requirement of all scientists and so this last point should be omitted.

 Your three first points to propose to enable open science more effectively are valid, however, you don’t circle back to these in your final discussion. May I suggest using instead the points that you end with, such as:

1. Finding a way to properly cite open codes or data available through open sources
2. Developing a reliable, robust tool to track utilization of open science (note: similar idea to 1, but goes one step further)
3. Universities need to support infrastructure to implement FAIR data principles

This way you end with developing the points that should enable open science.

 All these are suggestions. Should the authors decide to ignore them, it will not dampen my enthusiasm for this well-written manuscript.

I have read this submission. I believe that I have an appropriate level of expertise to confirm that it is of an acceptable scientific standard.

# Response

**Published:** 2017-05-17

**Authors:** Adina Howe

**Affiliations:** Iowa State University, USA

I would replace the words “the whole enchilada” under the headline “Open science requires significant Investment”. I would also replace “so on” in the same section.

 Modified as suggested.

 Instead of the headline “Broader impact of open science is uncertain” may I suggest something along the line of: “Open and broad communication could impact open science”.

 Modified the heading to “The Potential for Broader Impacts with Open Science”

 In the section “Open Science requires significant investment”, it is suggested that a key cost of open science is time, which is reasonable. However, one of the points is that “time to produce scripts with a sufficient level of robustness and documentation to be useful to others”; this point is less reasonable. I believe with or without open science, this should an integral requirement of all scientists and so this last point should be omitted.

 Modified this sentence to “time to produce scripts with a sufficient level of robustness and documentation to be **broadly** useful to others”.  We agree that there is a minimal requirement of reproducibility for all scripts.  However, our aim with this sentence was to convey that the impact of this e.g, documentation or code and its ability to be reproduced by a broad audience (e.g., the public vs. domain experts) requires significant and accountable requirements on time.  We feel like the inclusion of the “broadly useful” more appropriately captures our intent – thank you for your suggestion!

 Your three first points to propose to enable open science more effectively are valid, however, you don’t circle back to these in your final discussion. May I suggest using instead the points that you end with, such as:

1. Finding a way to properly cite open codes or data available through open sources
2. Developing a reliable, robust tool to track utilization of open science (note: similar idea to 1, but goes one step further)
3. Universities need to support infrastructure to implement FAIR data principles

Great suggestion.  Given the length of this article, we’ve provided some more directed heading to guide the conclusion, which are aligned with your suggestions.

 This way you end with developing the points that should enable open science.
